# Breast Mass Detection in Mammography Based on Image Template Matching and CNN

**DOI:** 10.3390/s21082855

**Published:** 2021-04-18

**Authors:** Lilei Sun, Huijie Sun, Junqian Wang, Shuai Wu, Yong Zhao, Yong Xu

**Affiliations:** 1 College of Computer Science and Technology, Guizhou University, Guiyang 550025, China; sunlileisun@163.com; 2 College of Computer Information and Engineering, Nanchang Institute of Technology, Nanchang 330044, China; sunhj6@sysu.edu.cn; 3 College of Computer Science and Technology, Harbin Institute of Technology (Shenzhen), Shenzhen 518055, China; wangjunqian@stu.hit.edu.cn (J.W.); shuaiwu9@gmail.com (S.W.); 4 School of Electronic and Computer Engineering, Shenzhen Graduate School of Peking University, Shenzhen 518055, China; zhaoyong@pkusz.edu.cn; 5 Shenzhen Key Laboratory of Visual Object Detection and Recognition, Harbin Institute of Technology (Shenzhen), Shenzhen 518055, China

**Keywords:** medical image processing, mammographic image, deep learning, breast mass detection

## Abstract

In recent years, computer vision technology has been widely used in the field of medical image processing. However, there is still a big gap between the existing breast mass detection methods and the real-world application due to the limited detection accuracy. It is known that humans locate the regions of interest quickly and further identify whether these regions are the targets we found. In breast cancer diagnosis, we locate all the potential regions of breast mass by glancing at the mammographic image from top to bottom and from left to right, then further identify whether these regions are a breast mass. Inspired by the process of human detection of breast mass, we proposed a novel breast mass detection method to detect breast mass on a mammographic image by stimulating the process of human detection. The proposed method preprocesses the mammographic image via the mathematical morphology method and locates the suspected regions of breast mass by the image template matching method. Then, it obtains the regions of breast mass by classifying these suspected regions into breast mass and background categories using a convolutional neural network (CNN). The bounding box of breast mass obtained by the mathematical morphology method and image template matching method are roughly due to the mathematical morphology method, which transforms all of the brighter regions into approximate circular areas. For regression of a breast mass bounding box, the optimal solution should be searched in the feasible region and the Particle Swarm Optimization (PSO) is suitable for solving the problem of searching the optimal solution within a certain range. Therefore, we refine the bounding box of breast mass by the PSO algorithm. The proposed breast mass detection method and the compared detection methods were evaluated on the open database Digital Database for Screening Mammography (DDSM). The experimental results demonstrate that the proposed method is superior to all of the compared detection methods in detection performance.

## 1. Introduction

Breast cancer has the highest mortality rate among all cancers, it greatly threatens the lives of women globally [1]. However, if getting aggressive treatments are conducted in the early stage of breast cancer, 90% of patients can be cured [2]. Therefore, how to accurately detect breast cancer in the early stages is greatly meaningful for cancer treatment. The molybdenum X-ray mammography is the most widely applied tool in breast cancer diagnosis due to it being less harmful to the patient, showing a good representation of breast mass characteristics and the fact it is low-cost. With the development of machine learning technology [3,4], using a computer to automatically diagnose breast cancer can improve diagnosis accuracy and save valuable medical resources.

**Figure 1:**
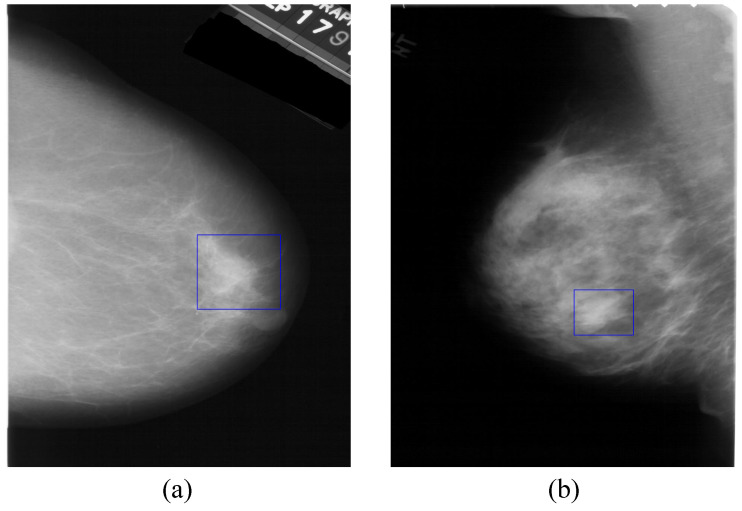
Molybdenum target mammograms. The regions of blue rectangles in (**a**,**b**) are breast masses.

The diagnosis of breast cancer on mammography contains two stages: breast mass detection and classification. The first stage is used to find the locations of all the suspected regions of breast masses in a mammographic image [5], and the second stage is used further to classify these suspected regions into breast mass and background categories. Breast mass detection plays a crucial role in diagnosis, the accuracy of breast mass detection greatly affects the performance of breast cancer diagnosis. Figure 1 shows two molybdenum target mammograms. The regions in the blue rectangles are breast masses. However, it is challenging work to detect breast masses in a mammographic image due to the blurry edge and complex texture of breast mass, etc. Kerhet et al. [6] converted the breast mass detection problem into a classification problem. They converted microwave breast sensing into probability mapping which illustrates a posteriori probability of tumor presence by the support vector machine (SVM) classification method, then located the breast mass according to the probability mapping. Kom et al. [7] enhanced mammographic images by a linear transformation filter, then segmented the breast mass in the enhanced image by a local adaptive threshold method. Xu et al. [8] segmented the breast mass by the improved method based on dynamic programming. The improved method generates the optimal weights of the cost components that are optimized by PSO [9].  Kuo et al. [10] enhanced the signal of the suspected regions of breast mass in the mammographic image in the time domain, then identified and located the breast mass by PSO. To enhance the regions of breast mass, StojiC et al. [11] enhanced the details on the mammographic image using the mathematical morphology method. Amutha et al. [12] enhanced the contrast of mammography using mathematical morphology. The region of breast mass always looks brighter than the neighboring regions due to the density of the breast mass region being higher than that of the surrounding tissues. Liu et al. [13] detected the breast mass using the image template matching method with a bright circular image template. The mathematical morphology method can highlight the region of interest in the image and guides people to focus on these regions, which are the objects we want to find with a high probability in the object detection task. Then, matching these regions using the image template matching method and identifying whether they are breast mass by a classifier. The process is similar to that of the physicians and radiologists glancing at the whole mammographic image to find the suspected regions of breast mass.

In recent years, machine learning technology, especially deep learning technology has been widely used in the field of computer vision [14], such as image object detection and image classification [15,16,17,18]. Compared with the conventional shallow learning methods or human-based feature extraction methods, CNN-based deep learning can adaptively extract the most discriminative features from the input images for different tasks and many reported experimental results have also demonstrated the superiority of deep learning in object detection and image classification tasks. In the field of object detection, CNN boasts many achievements. R-CNN [19] generates 2000 proposal regions by Selective Search [20] and normalizes these regions to a uniform size, then extracts the features from these regions via a CNN, and classifies these extracted features by an SVM and regresses the bounding box of the object by a feedforward network. Almasni et al. [21] detected and classified the breast mass by YOLO [22] on the mammographic image dataset Digital Database for Screening Mammography (DDSM) [23], the YOLO-based approach can detect and classify the breast mass simultaneously in one network. Kooi et al. [24] compared the detection performance of a CNN-based CAD system and the traditional CAD system which using hand-crafted image features on a large data set of around 45,000 images. The experimental results demonstrate that the CNN-based CAD system outperforms the traditional CAD system, and the CNN-based CAD shows a similar performance to that of the radiologists. To improve the diagnosis performance, Wu et al. [25] proposed a hybrid model which uses a two-stage architecture to diagnose breast cancer. The proposed method generates two heatmaps from each image by a sliding window as additional input views to a multi-view CNN-based classifier to improve the classification performance. The authors compared the performance of the proposed image-and-heatmaps ensemble method to humans with 12 attending radiologists, a resident and a medical student. The experimental results show that the proposed method is as accurate as the experienced radiologists on the same dataset.

Although there are many approaches to accomplish the detection task for mammography breast mass, the following problems still exist: First, there is no method is used to detect breast mass by simulating the human’s visual characteristics for object detection. The detection performance of breast mass can be improved significantly by simulating the human vision mechanism. Second, it is difficult to detect breast mass due to blurry borders, complex textures and the overlap of many kinds of human tissues in the mammographic image.

To solve the above problems, we propose a novel breast mass detection method in a mammographic image. Compared with the existing breast mass detection methods, the proposed method has the following advantages:

(1) For the blurry edge and complex texture of the breast mass, we highlight the suspected regions of breast mass and suppress the background by the eroding and dilating operations designed specifically for breast mass detection in this paper. We locate the suspected regions of breast mass in the processed mammographic image using the image template matching method. These matched regions will be further identified as breast mass or background by the following works.

(2) The suspected regions of breast mass cropped from the mammographic image are classified as breast mass or background by BD-CNN. We realized the detection task for breast mass and obtain a better detection performance in the mammographic image via the mathematical morphology method, image template matching method and a classification network.

(3) For the rough bounding box of the breast mass obtained from the mammographic image via the mathematical morphology and image template matching methods, we refined the bounding box of breast mass using PSO.

**Figure 2:**
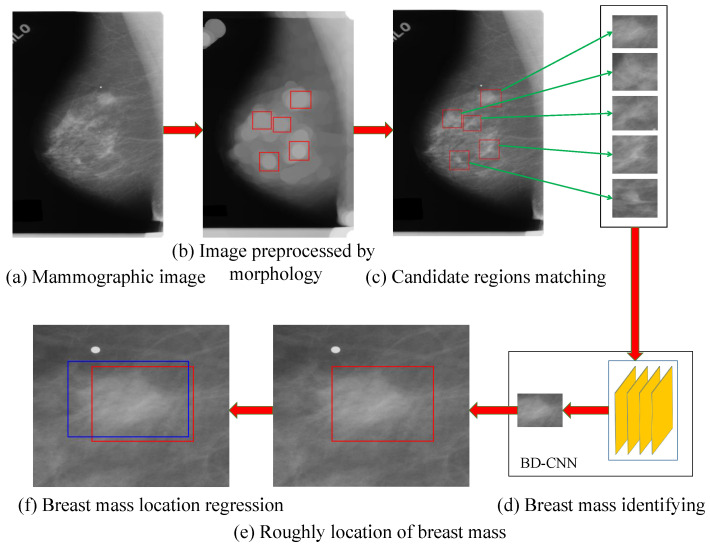
Flow chart of the breast mass detection method for the mammographic image. Where (**a**) is the mammographic image, (**b**)shows the matched regions on the processed mammographic image, (**c**) shows the suspected regions of breast mass generated using the mathematical morphology and image template matching methods on (**a**). (**d**) shows the process of breast mass identified using BD-CNN. The red rectangles in (**e**,**f**) are the regions of breast mass generated by the previous works. The blue rectangle in (**f**) is the refined bounding box of a breast mass using PSO.

The processing flow chart of the breast mass detection for a mammographic image proposed in this paper is shown in Figure 2.

The remainder of this paper is organized as follows. Section 2 introduces relevant works. Section 3 details the breast mass detection method of a mammographic image proposed in this paper. Section 4 conducts and analyzes the experiments. Section 5 concludes the breast mass detection method proposed in this paper.

## 2. Related Work

Over the past years, various image processing approaches have been proposed to improve the performance of image detection and classification. In this section, we introduce several image processing methods related to the method proposed in this paper. These methods are divided into two types: the mathematical morphology method and image template matching method.

The mathematical morphology method is used to process the mammographic image, which is beneficial to easily and effectively highlight the suspected regions of breast mass. There are several benefits of using the mathematical morphology method in a mammographic image: the noise can be removed, the suspected regions of breast mass can be highlighted and normalized to a circle shape, which is beneficial to improve the performance of the breast mass detection method. Eltonsy et al. [26] found that the regions of the breast mass are brightest in these concentric layers, they contain more potential information for the breast mass and the probability that the bright area is a breast mass is very high. They proposed a detection method based on a morphological model with concentric circles to detect the breast mass in the mammographic image. Because the gray-level values of the image pixels in each concentric layer are very similar, they realized breast mass detection by extracting different concentric regions using a predetermined threshold. To remove radiopaque artifacts, such as label text in the mammographic image,  Nagi et al. [27] transformed the mammographic image with grayscale into a binary [0, 1] using a global threshold with a value of T = 18. Then, obtaining the whole area of the breast by removing the isolated pixels and small objects such as labels text in the image with the binary format by a morphological operation. Ciecholewski et al. [28] proposed a segmentation method for the mammographic image. The proposed method is composed of two parts. In the first part, it reduces the noise and improves the contrast of the mammographic image via the mathematical morphological method. In the second part, it extracts microcalcification shapes in the processed mammographic image using watershed segmentation.

The image template matching method is used to find the most similar regions in the image to the template image. Tourassi et al. [29] built a databank for mammography ROI by the ground truth of breast mass from DDSM. The mutual information [30] is used as the similarity of the image template matching method. The similarities between the suspected region and all ROIs in the databank are calculated and rank-ordered and the detection result is obtained based on the best matches. Divyashree et al. [31] divided the breast mass detection task into several stages. They highlighted all the suspected regions of breast mass using the mathematical morphological method and enhanced these regions via the contrast limited adaptive histogram equalization (CLAHE) method [32]. Then, they detected the breast mass using a maximally stable external regions (MSER) [33] detector.  Lbachir et al. [34] segmented the regions of breast mass using the OST method [35]. Since these regions of breast mass segmented by OST are rough, they fine-turn the regions by the K-Means methods.

## 3. The Proposed Method

Matching the suspected regions of breast mass via the image template matching method on the processed mammographic image and identifying these regions by the classification method based on CNN can reach a satisfactory performance of breast mass detection. The method proposed in this paper contains four phases: the mammographic image processing based on the mathematical morphology method, the suspected regions of breast mass generation, the suspected region of breast mass identification and the bounding box of breast mass regression.

### 3.1. Processing of Mammographic Images by the Mathematical Morphology Method

The diagnosis process of breast cancer is first to roughly locate the suspected regions of breast mass by scanning the whole mammographic image, and then further identify these suspected regions as breast mass and background categories carefully. We highlight the suspected regions of breast mass in the mammographic image using the mathematical morphology method.

We divide the process of mammographic image using the mathematical morphology method into two stages: image eroding and image dilating. In the image eroding stage, an eroded kernel with small size is used to eliminate the meaningless small targets, such as noise pixels, and highlight the high-power regions in the mammographic image. In the image dilating stage, the high-power region is normalized to an approximate circular region with a fixed size by a dilating kernel with a large size. This is beneficial to match the suspected regions of breast mass more accurately by the image template matching method. Because breast mass is almost an ellipse or oval in shape, we used an ellipse eroding kernel with a size of 7 × 7 pixels and an ellipse dilating kernel with a size of 50 × 50 pixels to process the mammographic image to retain the morphological information of the breast mass.

### 3.2. The Generation Model for Candidate Region of Breast Mass

The image template matching method for breast mass detection flips all the potential breast mass regions from top to bottom and from left to right on the image *I* by an image template *T*, and calculates the similarity between *T* and the potential mass regions. The regions with high similarity to *T* are considered as the suspected regions of a breast mass.

The image template matching method applies the image template to the image in a certain order to deal with all the image patches and measures the Euclidean distance between the image template and these patches. The Euclidean distance between the image template and an image patch can be formulated as

(1) $$D\left(i,j\right)=\sum_{m=1}^{M}\sum_{n=1}^{N}{\left[P\left(m,n\right)-T\left(m,n\right)\right]}^{2},$$

where D(i,j) is the Euclidean distance between the image template and the image patch whose center of gravity is located at (i,j). *M* and *N* are the width and height of the image template, respectively. The width and height of the image template are 68 pixels and 60 pixels, respectively. P(m,n) and T(m,n) are the pixel values of the image template and an image patch whose center of gravity is located at (m,n), respectively.

**Figure 3:**
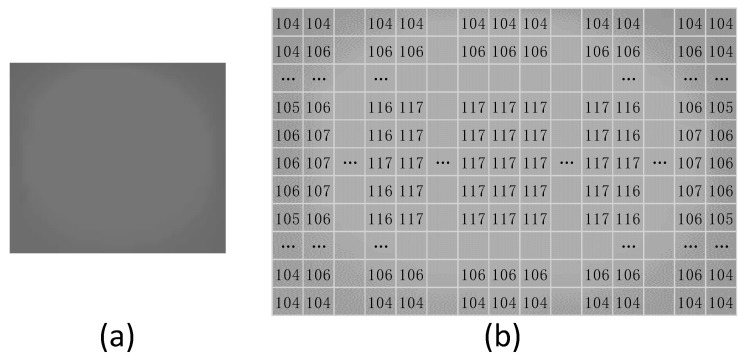
The circular image template of breast mass for image template matching method. Where (**a**) is the template image used to match the suspected regions of breast mass in the processed image. The pixel values of the template image of breast mass are shown in (**b**).

To generate a better breast mass template image, we processed a mammographic image which contains a typically breast mass by the mathematical morphology method, then cropped and saved the mass region in the processed mammographic image as the breast mass template image. As shown in Figure 3, typical pixel values are 117, 116 and 104 in (b) are the pixel value in the breast mass template image. The designed image template presents a circle, the pixel values in the central region are 117, and the pixels are decreasing toward the edge and the outermost pixels are 104.

### 3.3. Candidate Regions Identification Model

**Figure 4:**
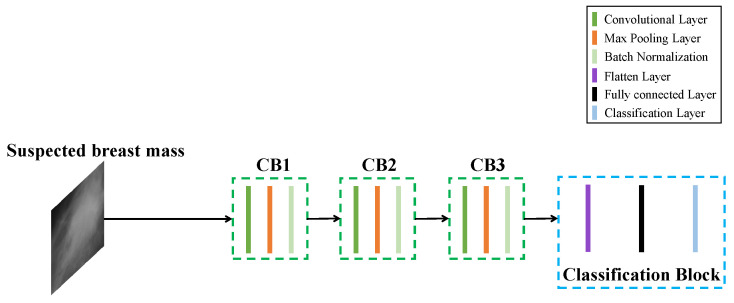
Architecture of DB-CNN.

After obtaining the suspected regions of breast mass from the mammographic image by the previous works, we identify these suspected regions by a classification method based on CNN. An image classification method for breast mass detection based on CNN (BD-CNN) is proposed to classify these suspected regions into breast mass and background categories. The architecture of BD-CNN is shown in Figure 4.

BD-CNN contains an input, output, three convolutional blocks (CB), a flatting layer, a fully connected layer and a classification layer. Each CB contains a convolutional layer, a max-pooling layer, a Batch Normalization (BN) [36] and ReLU activation function. There are 128 convolutional kernels in each CB, and the size of convolutional kernels used in CB1, CB2 and CB3 are 5 × 5, 3 × 3 and 3 × 3, respectively. BD-CNN receives the image of the suspected breast mass region, which is resized to 200 × 200 pixels from the input and generates the feature maps by three CBs. Then, these feature maps are flattened by a flatting layer and the discriminant features are further extracted by a fully connected layer containing 1024 nodes. Finally, the output of the fully connected layer is classified into breast mass and background categories by a classification layer with softmax.

### 3.4. Regression Model for the Location and Bounding Box of Breast Mass

**Figure 5:**
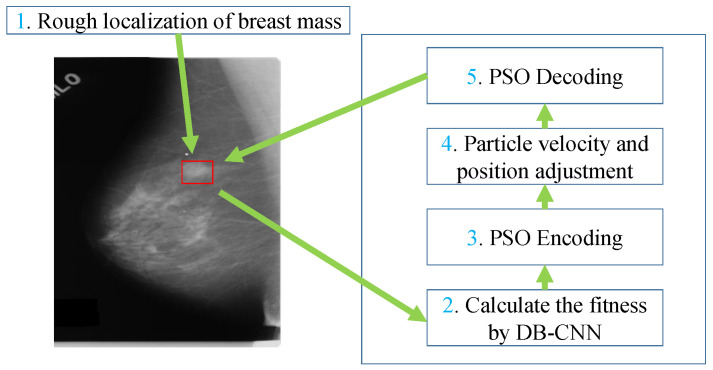
Flow chart of breast mass bounding box regression using PSO.

The suspected regions of breast mass generated by the mathematical morphology and the image template matching methods means that the bounding box of breast mass are rough. To solve the problem of the rough bounding box of breast mass in the mammographic image, the PSO algorithm is used to search a location and bounding box better to match the ground truth of the breast mass because it can exploit the potential solution by a global random research mechanism. The flow chart of breast mass bounding box regression conducted using PSO is shown in Figure 5. The rough bounding box of breast mass from the mammographic image is used as the initialization of the regression model. The BD-CNN is used as the fitness function of PSO to predict the probability that the region is a breast mass, and the region is optimized by the PSO based on the probability. PSO Encoding encodes the bounding box of breast mass on the mammographic image into a particle feature, i.e., the center coordinate x and y, width and height. PSO Decoding transforms the particle feature into the bounding box of a breast mass on the mammographic image. Steps 2 to 5 are the iterative optimization processes for the regression of the breast mass bounding box in the mammographic image.

The position of the *i*-th particle is defined as $X_{i}=\left(x_{p}^{i},y_{p}^{i},w_{p}^{i},h_{p}^{i}\right)$, the velocity of the *i*-th particle is defined as $V_{i}=\left(x_{v}^{i},y_{v}^{i},w_{v}^{i},h_{v}^{i}\right)$. $x_{p}^{i}$ and $y_{p}^{i}$ are the center of the *i*-th potential breast mass, $w_{i}$ and $h_{i}$ are the width and height of the *i*-th potential breast mass, respectively. $x_{v}^{i}$ and $y_{v}^{i}$ are center of the velocity of the *i*-th particle, $w_{v}^{i}$ and $h_{v}^{i}$ are the width and height of the velocity of the *i*-th particle, respectively. Each particle calculates the fitness by the corresponding object function. In our work, the fitness is generated by BD-CNN. In addition, every particle knows its best fitness pbest at present. $pbest_{i}$ can be regarded as the search experience of the *i*-th particle. Every particle knows the best global fitness gbest found by all of the particles in the entire population so far. gbest can be regarded as the best research experience of the peers. The initial value of $pbest_{i}$ is set as the position of the *i*-th particle. The initial value of gbest is the maximum pbest of all the particles. The next motion of a particle is determined by its experience and the experiences of all of the peers, the motion can be formulated as

(2) $$\begin{array}{c} V_{i}=V_{i-1}+c_{p}\times rand\left(\right)\times \left({pbest}_{i}-x_{i-1}\right)\\ +c_{g}\times rand\left(\right)\times \left(gbest-x_{i-1}\right), \end{array}$$

(3) $$x_{i}=x_{i-1}+V_{i},$$

where rand() is used to generate a random number between (0, 1). $c_{p}$ and $c_{g}$ are the weights of the search experience of the *i*-th particle and all the particles, respectively. The values of $c_{p}$ and $c_{g}$ are set to 0.5 in this paper. The bounding box of the breast mass refinement algorithm based on PSO as shown Algorithm 1.
**Algorithm 1.** PSO for breast mass bounding box refinement. **Input:** Number of particles, maximum iteration number, classification model of breast mass based on CNN: Prediction_Model, rough bounding box of breast mass X(x,y,w,h) **Output:** $best_{X}$  **for**
i=1 to number of particles **do**    $X_{i}=X$    $V_{i}=\left[0,0,0,0\right]$    $pbest_{i}=Prediction\_Model\left(X_{i}\right)$  **if**
$gbest<pbest_{i}$
**then**    $gbest=pbest_{i}$  $best_{x}=X$  **for**
p=1 to Maximum iteration number **do**    **for**
i=1 to Number of particles **do**        1. Update $V_{i}$        $V_{i}=V_{i}^{\left(-1\right)}+c_{p}\times rand\left(\right)\times pbest_{i}-X_{i}^{\left(-1\right)})+c_{g}\times rand\left(\right)\times \left(gbest-X_{i}^{\left(-1\right)}\right)$        2. Update $X_{i}$        $X_{i}=X_{i}^{\left(-1\right)}+V_{i}$        3. Get fitness from $X_{i}$ by Prediction_Model        $fitness=Prediction\_Model(X_{i})$        4. Update $pbest_{i}$        **if**
$pbest_{i}<fitness$
**then**           $pbest_{i}=fitness$        5. Update tbest and $best_{x}$        **for**
j=1 to number of particles **do**           **if**
$gbest<pbest_{j}$
**then**               $gbest=pbest_{j}$               $best_{x}=X_{i}$

## 4. Experiments

We evaluate the proposed breast mass detection method for the mammographic image on DDSM. In this section, we first describe the DDSM mammographic image dataset and experimental setting. Then, we analyze the experimental results of the proposed method and the state-of-the-art breast mass detection methods for the mammographic image on DDSM in detail. Finally, the result of the bounding box regression of breast mass is illustrated using PSO.

### 4.1. Dataset and Experimental Setting

In this paper, a subset containing 439 mammographic images is selected from the DDSM to evaluate the different breast mass detection methods in the mammographic image. In our experiments, simple random sampling is performed to divide the samples in the subset into training and test datasets. The training and test datasets contain 70% and 30% of the samples, respectively. There are 307 mammographic images in the training dataset and 132 mammographic images in the test dataset. An unbiased estimate of the performance of the method proposed in this paper was obtained through the use of 5-fold cross-validation. The averages of five folds are used to evaluate the performance of these methods.

All of the mammographic images in the training and test datasets are resized to 1500 × 2000 pixels. All of the training samples of suspected breast mass are resized to 200 × 200 pixels. The weights of BD-CNN are randomly initialized, and the value of the learning rate is set to $10^{-4}$. The training process of BD-CNN is terminated if the number of training epochs reaches 200 or the training accuracy equals 100%. All CNN-based breast mass detection methods mentioned in this paper are optimized by the Stochastic Gradient Descent method, and these methods are implemented based on Python 3.7.3 and PyTorch 1.1.0, and evaluated in the following environment: Ubuntu 16.04, Intel(R) Xeon(R) CPU E5-2640 v4, RAM 256G and NVIDIA GeForce GTX 1080 Ti GPU.

### 4.2. Accuracy Comparison and Analysis

**Figure 6:**
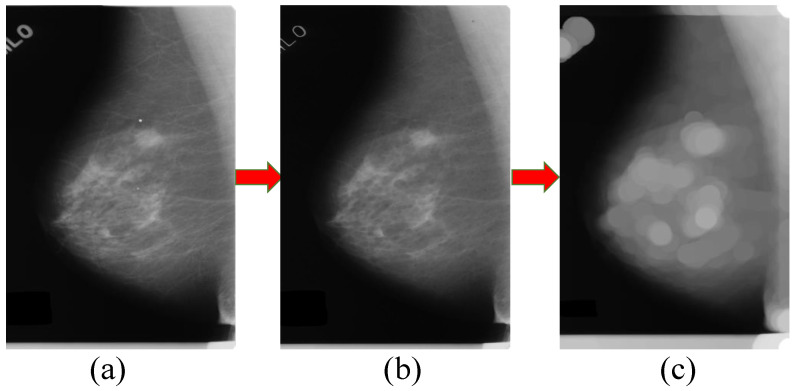
Processing by the mathematical morphology method. Where (**a**) is the mammographic image and there is some noise in the image. (**b**) is the image after the eroding operation on the mammographic image (**a**). Part (**c**) is the image after the dilating operation on image (**b**).

All samples in the training and test subsets are processed using the mathematical morphology method. In the first stage of the mathematical morphology method, a 7 × 7 pixel kernel is used to deal with the mammographic image. As shown in Figure 6, compared with the image (a), image (b) contains less noise and we can see the high-power regions in (b) more clearly than the mammographic image (a). To facilitate the extraction of the suspected regions of breast mass, a dilating operation with an ellipse dilating kernel with a size of 50 × 50 pixels is used to process the image after the eroding operation. We can find that the image in (c) is normalized the high-power regions into circular regions on image (b); it is easy to match using the image template matching method with a circular image template.

**Figure 7:**
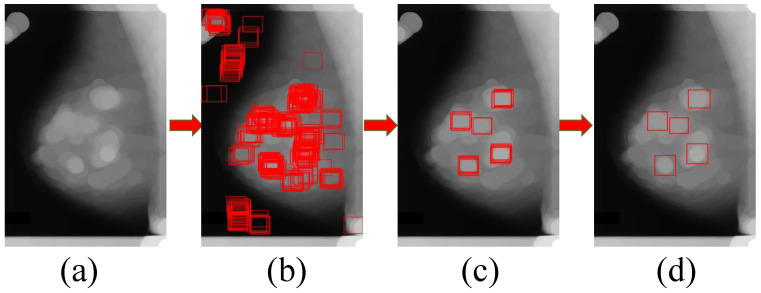
Process of breast mass candidate bounding box generation. Where (**a**) is the processed image using the mathematical morphology method, and (**b**) shows the matching results of suspected regions using the image template matching method with our breast mass image template on image (**c**,**d**) contains fewer suspected regions with a higher matching degree.

After obtaining the image with obvious highlighted circular regions, we match the suspected regions of a breast mass by the image template matching method. As shown in Figure 7, the image in (b) illustrates that some circular regions in image (b) are matched, but most of these matched regions are meaningless. For resolving the problem of the meaningless regions, we filter out the regions with a high matching degree. Compared with image (b), image (c) contains fewer suspected regions with a higher matching degree, which is beneficial to reduce the suspected regions with a lower probability of being a breast mass. For obtaining more precise suspected regions and reducing meaningless regions, we combine and eliminate these regions by their Intersection over Union (IoU). In particular, the two bounding boxes will merge if the IoU of them is greater than 0.3. As shown the image (d) in Figure 7, we obtain five circular regions from the regions overlapping each other in image (c) using the merging method.

**Figure 8:**
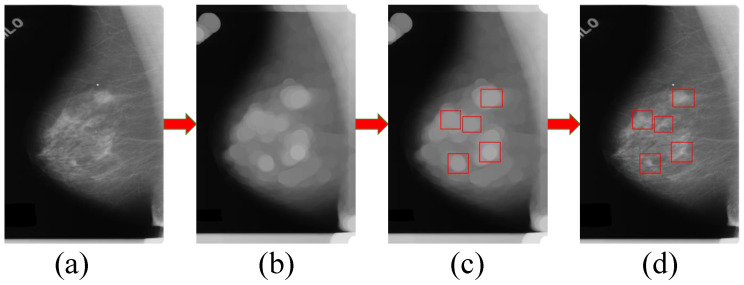
Process of candidate region generation for breast mass in a mammographic image by the mathematical morphology method and image template matching method. (**a**) is a mammographic image, and (**b**) is the processed image using the mathematical morphology method, (**c**,**d**) show the matched suspected regions of breast mass on the processed image and mammographic image, respectively.

After obtaining the circular regions in the processed image by the mathematical morphology and image template matching methods, we crop the suspected regions of breast mass from the mammographic image. As shown in Figure 8, we match these regions using the image template matching method with an ellipse dilating kernel with a size of 50 × 50 pixels. The regions enclosed by the red rectangles in the image (d) are the suspected regions of breast mass.

**Figure 9:**
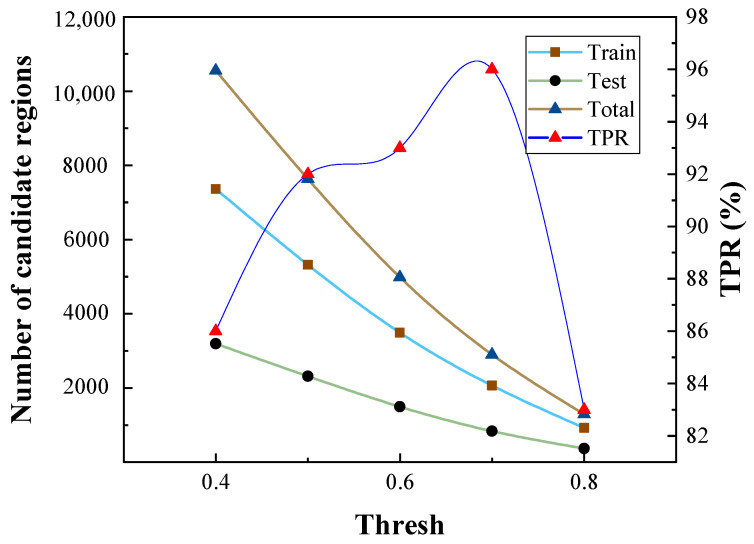
Comparison of the detection performance of the proposed method under different thresholds for matching degree.

**Figure 10:**
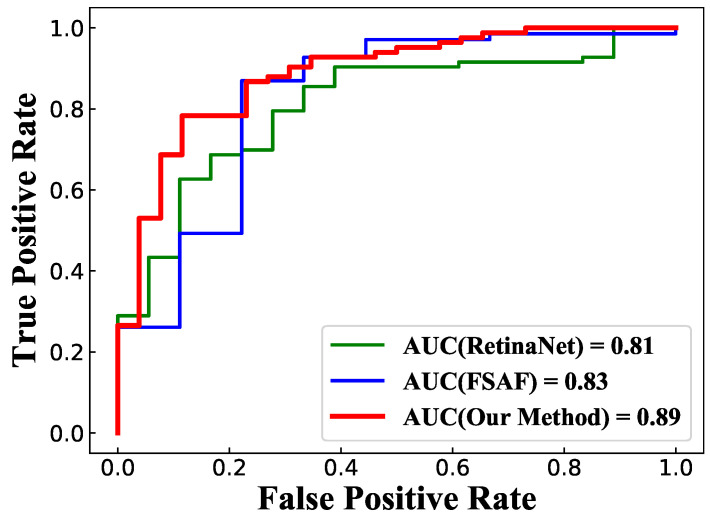
ROC curve of our method and the compared deep learning detection methods.

Different thresholds of the image template matching cause different numbers of suspected regions and have a great influence on the detection results. As shown in Figure 9, when the threshold is 0.4, there are 10,552 suspected regions cropped from mammographic images. The threshold is set to 0.7 in this paper and there are 2065 suspected regions (i.e., 307 breast masses and 1758 background) that are used as the training dataset and 836 suspected regions (i.e., 132 breast masses and 704 background) that are used as the test dataset. The experimental results of the proposed method and the state-of-the-art detection methods for the mammographic image are listed in Table 1 and Figure 10. Compared with traditional breast mass classification methods, deep learning can automatically extract discriminative features from mammographic images and avoid the problem of poor discriminative ability of features using the manual design feature extraction method. We cited the experimental results in the works of literatures for Eltonsy [26], Sampat [37], Wu [38], Junior [39], Liu [40] and Cao [41] in Table 1. RetinaNet [42], FSAF [43], Foveabox [44] and our method in Table 1 are evaluated on the subset used in this paper. The benefit from the proposed method simulates the breast mass detection process of radiologists via the multi-stage method, meaning it can detect the breast mass in a more intelligent way. The proposed method achieves the highest True Positive Rate (TPR) and the lowest False positives Per Image (FPI), performing better than all of the compared breast mass detection methods.

**Figure 11:**
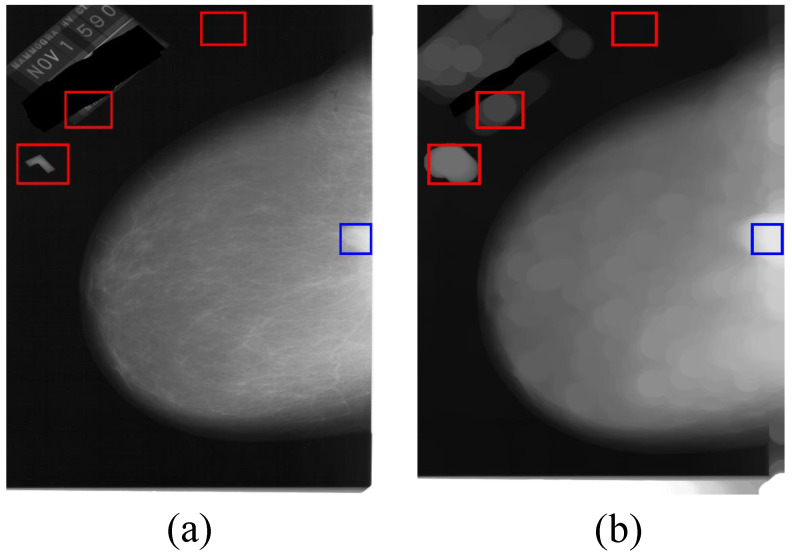
The mismatched breast mass. Where (**a**) is the mammographic image, (**b**) is the processed image of (**a**) by the mathematical morphology and image template matching methods.

Some of the regions of breast mass cannot be matched in the mammographic image due to the fact that they are not circular regions and the image template matching method is hard to match the non-circular region by our proposed image template. As shown in Figure 11, the red rectangles in (a) and (b) are the matched regions, the blue rectangles are the ground truth of the breast mass. The image template matching method does not match the breast mass because the shape of the breast mass in the processed image is not an ellipse.

**Figure 12:**
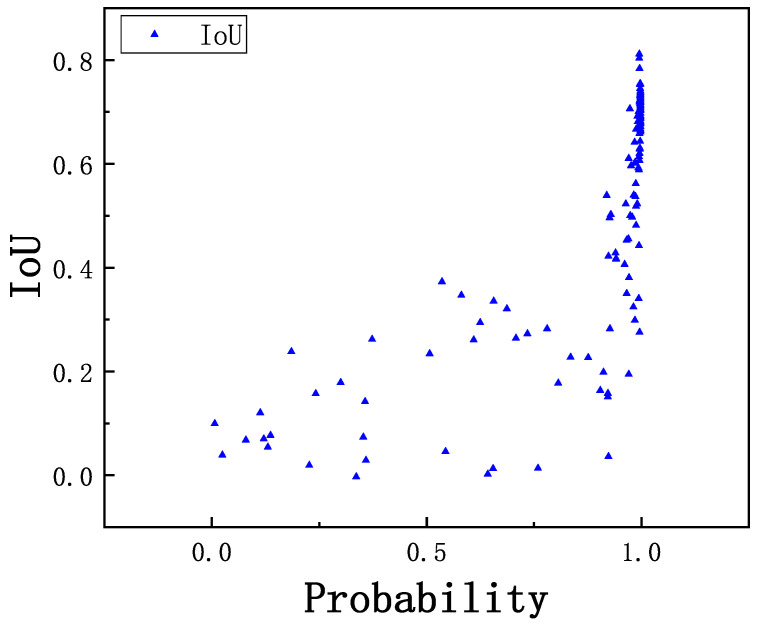
Data distribution of classification accuracy and IoU.

The bounding box of breast mass is a rough location generated by the image template matching method. In our experiment, the number of particles in the particle swarm is set to 20, and the maximum search epoch is set to 20. As shown in Figure 12, the data on the horizontal axis are the classification accuracies of breast mass and background obtained by BD-CNN. The data on the vertical axis are the IoU of the predicted bounding box and the corresponding ground truth. It illustrates that the higher the classification probability of breast mass and background by the BD-CNN, the higher the trend of IoU to a higher value. From the data distribution of the classification probability and IoU in Figure 12, it can be concluded that improving the IoU by improving the classification probability is a feasible method and can achieve satisfactory bounding box regression performance.

**Figure 13:**
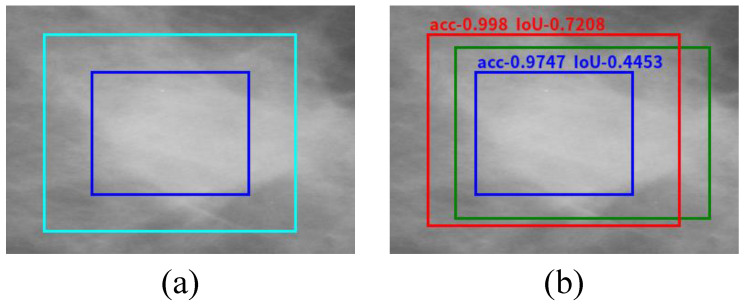
Optimization of breast mass bounding box. (**a**) shows the rough bounding box of the breast mass generated by the image template matching method (blue rectangle) and the search range for the effective resolution by PSO, respectively. (**b**) shows the regressed bounding box of the breast mass (red rectangle).

For attaining a better probability of breast mass detection by BD-CNN, we use PSO to optimize and search the feasible resolution. As shown in Figure 13, the blue rectangles in image (a) and image (b) are the rough bounding boxes generated by the image template matching method from the processed image. The cyan rectangle in image (a) is the search range for the effective resolution by PSO. The cyan rectangle is expanded from the rough bounding box with a blue color. In particular, the cyan rectangle is 30% of the height of the upward and downward expansion, and 30% of the width of left and right expansion. The green rectangle in image (b) is the ground truth of breast mass, and the red rectangle is the optimal matching result regressed using PSO. Image (b) illustrates that the bounding box of breast mass can be refined and a higher IoU can be achieved using PSO.

## 5. Conclusions

In this paper, we proposed a novel breast mass detection method that integrates the mathematical morphology method, image template matching method, BD-CNN and the regression model of breast mass bounding box based on PSO. The proposed detection method generates the suspected regions of breast mass by exploiting the mathematical morphology operations and the image template matching means. These operations extract more effective regions by simulating the process of human detection for these suspected regions of breast mass. Then, identifying the breast mass by classifying the suspected regions into breast mass and background categories. We use PSO to regress the bounding box to obtain a more suitable bounding box of breast mass. We evaluated the detection performance of the proposed method via experiments on the well-known mammographic image dataset DDSM and compared it with the state-of-the-art breast mass detection methods. The experimental results demonstrate that the proposed method outperforms all of the compared state-of-the-art breast mass detection methods. 

## Figures and Tables

**Table 1:** Comparison of the detection performance of the proposed method and the state-of-the-art methods on DDSM.

Method	Tradition /Deep	TPR (%)	Accuracy (%)	Precision (%)	Recall (%)	F1 Score (%)	FPI	Year
Eltonsy [26]	Tradition	92.1	-	-	-	-	5.4	2007
Sampat [37]	Tradition	88	-	-	-	-	2.7	2008
Wu [38]	Deep	81	-	-	-	-	1.1	2018
Junior [39]	Tradition	91.63	-	-	-	-	0.86	2019
Liu [40]	Deep	95	-	-	-	-	4.4	2020
Cao [41]	Deep	94.3	-	-	-	-	0.599	2020
RetinaNet [42]	Deep	91.95	88.41	56.39	91.53	69.79	1.18	2018
FSAF [43]	Deep	85.05	63.66	26.74	85.38	40.73	1.04	2019
Foveabox [44]	Deep	89.65	76.82	37.66	89.23	52.96	1.18	2020
Our method	Deep	96	85.82	50.81	95.38	66.31	0.53	-

## Data Availability

http://www.eng.usf.edu/cvprg/Mammography/Database.html accessed on 26 March 2021.
